# Ecosystem Services Linked to Extensive Sheep and Goat Farming in Mountain Areas: A Global Literature Analysis Using Text Mining and Topic Analysis

**DOI:** 10.3390/ani15030350

**Published:** 2025-01-25

**Authors:** Riccardo Primi, Gloria Bernabucci, Chiara Evangelista, Paolo Viola, Pedro Girotti, Raffaello Spina, Silvia Compagnucci, Bruno Ronchi

**Affiliations:** 1 Department of Agriculture and Forest Sciences (DAFNE), University of Tuscia, 01100 Viterbo, Italy; gloria.bernabucci@unitus.it (G.B.); p.viola82@unitus.it (P.V.); pedro.girotti@unitus.it (P.G.); raffaello.spina@unitus.it (R.S.); silvia.compagnucci@unitus.it (S.C.); ronchi@unitus.it (B.R.); 2 Department for Innovation in Biological, Agro-Food and Forest Systems (DIBAF), University of Tuscia, 01100 Viterbo, Italy; chiara.evangelista@unitus.it

**Keywords:** small ruminants, mountain regions, extensive farming, ecological services, grazing

## Abstract

This study investigates the ecosystem services (ES) associated with extensive sheep and goat farming in mountainous regions using advanced text mining (TM) and topic analysis (TA) methodologies. Mountain ecosystems are ecologically and socio-economically significant yet face challenges such as rural abandonment and climate change. The analysis of 135 scientific publications from 2009 to 2024 highlights the vital role of small ruminant farming in preserving biodiversity, maintaining cultural heritage, and providing sustainable livelihoods. Results identify eight key themes: ecosystem conservation; grazing effects on biodiversity; dimensions of sustainability; climate; people engagement; grazing and soil; transhumance; policies; and strategies. Findings underscore that small ruminant farming not only supports provisioning, regulating, and cultural services but also reveals ecosystem disservices (EDS) like soil erosion and biodiversity loss under poor management. Effective governance and adaptive management are crucial in integrating tools such as payment for ecosystem services (PES), certification systems, and advanced technologies. This study highlights knowledge gaps, including limited research on EDS and the need for standardised frameworks to assess ES. Future research should prioritise innovative technologies, region-specific grazing practices, and participatory governance to enhance the sustainability of these systems.

## Simple Summary

Mountain ecosystems, home to around one billion people globally, rely heavily on sheep and goat farming, which plays a vital role in sustaining local economies. These animals, well-adapted to harsh conditions, provide high-quality goods such as meat, milk, and wool. Recent research has increasingly focused on the ecosystem services that small ruminants in mountain systems deliver. Using text mining and topic analysis, this study explored the literature and identified eight key themes: ecosystem conservation; grazing effects on biodiversity; sustainability; climate impacts; people engagement; grazing and soil health; transhumance; and related policies. While scientific interest in small ruminant farming’s role in promoting sustainability, biodiversity conservation, and rural livelihoods has grown significantly in the past 15 years, critical gaps remain. Insufficient attention has been given to ecosystem disservices, and the lack of standardised frameworks for evaluating ecosystem services limits the ability to compare findings globally. Future research must prioritise the development of universal assessment frameworks and the integration of advanced tools such as artificial intelligence and remote sensing to improve monitoring and management. Collaborative, interdisciplinary approaches will be essential to optimising the benefits of small ruminant farming while addressing its challenges, ultimately supporting the resilience and sustainability of mountain ecosystems in the face of evolving global pressures.

## 1. Introduction

Mountain ecosystems are unique and complex environments characterised by their high altitudes, steep slopes, and significant climatic variations. Covering approximately 25% of the world’s land area, these regions host nearly one billion people and play an essential role in providing a set of Ecosystem Services (ES) essential for humans [1,2,3]. The diverse terrain and altitude create distinct climatic zones that can change drastically also over short distances, supporting a wide range of flora and fauna and making mountains hotspots for biodiversity [4,5]. Human activity has shaped mountain environments for thousands of years, turning wild ecosystems into agro-pastoral systems adapted to the unique environmental conditions of these regions [6,7]. Low temperatures, short growing seasons, limited sun exposure, challenging topography, and poor organic soil make most agricultural practices problematic or impossible, leading to the predominance of extensive livestock systems [5,8,9].

Small ruminants play a crucial role in this context due to their exceptional capacity to adapt to hostile environments, enabling productive use of areas otherwise unsuitable for crop cultivation [10,11]. Their lower feed and capital requirements, along with their ability to utilise a wide range of feedstuffs and marginal land, make them a suitable livelihood source for smallholders and rural communities [12].

**Figure 1:**
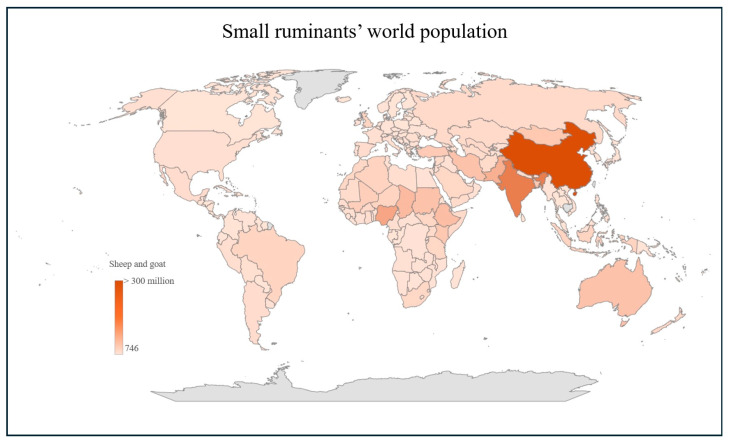
Worldwide distribution of small ruminants by country, ranging from 746 to over 300 million heads, represented by colour gradients (License: CC-BY-4.0) [13].

Small ruminants’ world population is estimated at 2.467 billion heads, consisting of 1.322 billion sheep and 1.145 billion goats [13]. As shown in Figure 1, Asia hosts the largest share, with 45.8% and 50.7% of sheep and goats respectively, followed by Africa (31.7% of sheep; 44.2% of goats), Europe (9.1% of sheep; 1.3% of goats), the Americas (6.2% of sheep; 3.4% of goats), and Oceania (7.2% of sheep; 0.4% of goats) [13]. However, no data are available on the specific population of small ruminants reared in mountainous areas.

In such mountain regions, characterised by forests, shrubs, and meadows, well-managed pastoral activities can serve as valuable tools for aesthetic and functional landscape preservation, fire prevention [14], and grassland biodiversity maintenance. These activities, which can be referred to as ES, also contribute to the economic well-being of mountain communities by providing niche products, mainly high-quality foods, to the market [15,16]. Despite the importance of small ruminants in supporting Mountain Ecosystem Services, a detailed understanding of their distribution, management practices, and ecological impacts remains fragmented.

Advanced methodologies such as text mining (TM) and topic analysis (TA) offer a promising approach to bridge this knowledge gap by systematically analysing the growing body of the scientific literature, uncovering patterns, and identifying key themes related to small ruminant systems and their roles in mountain areas. Methods like TM and TA offer effective, unsupervised alternatives, enabling the automatic identification of recurrent topics and latent themes within large datasets [17]. These methods can extract valuable insights, summarise data into visual formats, and reveal associations between concepts, creating structured maps of textual knowledge [18,19]. Text mining not only assists researchers in analysing extensive document collections but also provides valuable insights for stakeholders in the livestock sector by uncovering critical patterns and relationships that inform decision-making [20]. For these reasons, TM techniques have been increasingly utilised in the scientific literature analyses to identify key themes and potential directions for future research in fields such as livestock management. Recently, several papers have been published on various topics within the livestock sector, ranging from precision livestock farming [21] and automated milking systems [22] to the well-being of different species [23,24] and enteric methane emissions [25]. While studies have examined the ES of mountain livestock farming [26] and ES more broadly [27], there remains a significant gap in targeted knowledge regarding the specific ES provided by small ruminant farming in mountain areas. This article aims to address this gap by exploring topics related to the ES of small ruminants in mountainous regions through TM and TA techniques. Specifically, it seeks to describe the evolution of the literature, analyse geographical distribution, identify key research topics, and highlight existing gaps in knowledge.

## 2. Materials and Methods

### 2.1. Data Collection

**Figure 2:**
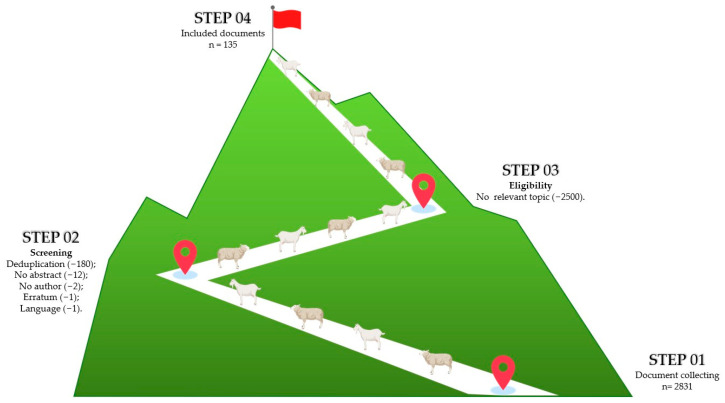
Preprocessing of the scientific literature on raw data to obtain the final dataset, with all the steps, including information about exclusions.

Utilising Scopus^®^, the Elsevier© abstracts and citation database, a search protocol was developed to identify peer-reviewed documents related to ES associated with extensive sheep and goat farming in mountain areas, characterised by grazing-based practices. This search was performed on 11 October 2024, and the year range was set from 1980 onwards. The keywords set for the search were “Ecosystem services” AND “mountain”, “Ecosystem services” AND “ruminants” AND “mountain”, “Ecosystem services” AND “small ruminants”, “Ecosystem services” AND “ruminants”, “Ecosystem services” AND “sheep” OR “goat”, “Ecosystem services” AND “pastoral”, “Ecosystem services” AND “mountain livestock”. English language and selected subject areas were set before starting the search. The selected subject areas include Environmental Science, Agricultural and Biological Sciences, Social Sciences, Earth and Planetary Sciences, Economics, Econometrics and Finance, Veterinary Sciences, Biochemistry, Genetics and Molecular Biology, Arts and Humanities, and Multidisciplinary Studies. Each sequence of keywords produced a certain number of records, which were then downloaded (Table 1). The data extracted from these records were consolidated and organised in a commercial spreadsheet (Microsoft Excel^®^, version 16.0, Redmond, WA, USA) for further analysis. The final spreadsheet comprised each record as a row, with associated information organised into columns. The collected information comprised the title, authors, affiliations, abstract, year of publication, type of record (e.g., research article or review), and publication source (e.g., journal title). A total of 2831 documents were extracted. Reasons for subsequent exclusion from the database were the following: no author available; no abstract available; and no keywords available. In addition, a screening of all documents was performed to delete the duplicates. Additionally, the authors manually reviewed the database to remove abstracts considered off-topic, excluding documents focused on forest ES, other animal species, or environments unrelated to the study of ES linked to extensive sheep and goat farming in mountain areas. The final dataset consisted of 135 documents (Table S1). Details regarding the exact number of records downloaded from each research string, the eligibility procedure, and the initial screening are displayed in the flow chart (Figure 2). Each record was associated with a nationality that was extrapolated from the country or geographical region of the affiliation of the corresponding author and, where not present, of the first author. Descriptive statistics on year, country of publication, and type of record were performed to profile the scientific corpus using the R program (v. 4.3.1) [28].

### 2.2. Text Mining

Text mining analysis was conducted to identify the key terms within the data corpus. This technique transforms textual information into numerical data, examining word frequency distributions and the relationships between the most used words [29].

To preprocess the text data, we followed the three-step approach outlined by Sebastiani [30], which includes tokenisation, filtering, and stemming. Initially, all words were converted to lowercase, and stopwords, punctuation, whitespace, and numerical digits were removed during the tokenisation and filtering stages. In addition, terms that were frequently used during the paper selection process (such as ecosystem services, mountain, ruminants, small ruminants, sheep, goat, pastoral, and livestock) were excluded from the dataset to prevent the over-representation of common words and ensure the analysis maintained its discriminative power. In the final phase, stemming was applied to reduce words to their root forms, standardising their representation and improving the accuracy of word frequency analysis. The words were subsequently organised into a document-term matrix (DTM), with terms represented as columns and documents as rows. To account for the importance of terms in relation to their frequency, a term frequency–inverse document frequency (TF–IDF) technique was applied. This method, as described by Salton and Buckley [31], weighs the frequency of words in abstracts relative to their commonality across all abstracts. The aim is to highlight terms that are more significant within the entire document collection. This initial step in TM formed the basis for building a document corpus and transforming it into a DTM, which then served as input data for TA.

The statistical analysis was performed using R 4.3.1 [28] with the following libraries: “tm”; “stringr”; and “snowball3”.

A cloud of the most relevant words was also created [32] in which, a bigger character size indicates a higher TF–IDF value. VOSviewer v. 1.6.19 [33] was used to perform the co-occurrence analysis of terms, as well as to analyse the connections between the authors’ countries. This software tool specialises in constructing and visualising bibliometric networks and offers TM functionality to generate co-occurrence networks of significant terms extracted from the scientific literature.

### 2.3. Topic Analysis

Topic analysis is aimed at exploring and interpreting document content to extract meaningful insights from unstructured text data. In this study, Latent Dirichlet Allocation (LDA) [17] was employed in the abstract dataset. LDA is a Bayesian probabilistic method that identifies latent thematic topics across a document corpus based on the co-occurrence of words within texts. This approach assumes the presence of the *K* latent topics shared across the documents [34]. The number of topics must be predefined; however, determining the optimal number remains a challenging task. If too few topics are chosen, the complexity of the corpus may not be properly captured. Conversely, selecting too many topics can lead to difficulties interpreting the results [29]. To address this issue, multiple tests with varying topic counts (e.g., five, six, seven, eight, and nine) were conducted. Eight topics were selected as the optimal number, as they produced the most coherent results, with each topic representing at least 5% of the documents, ensuring a sufficient sample size for analysis. Each topic was visualised as an individual bar histogram, illustrating the probability of the top 10 terms within the topic, as determined by beta values. Beta values represent the likelihood that specific words are associated with a given topic. After finalising the topic count, researchers assigned preliminary labels to the topics based on the top 10 terms and the content of the articles associated with each topic. These labels were further refined through group discussions to achieve a consensus on the final designations. Subsequently, each topic was categorised based on its role as either contributing to ES or EDS.

Descriptive statistics, including the number of publications and the initial year of publication for each topic, were analysed using an Excel spreadsheet. Additionally, the most frequent country associated with each topic was identified. The LDA algorithm, with the Gibbs sampling option, was implemented using the “topicmodels” R package [35]. To visualise the 10 most frequent words in each topic, along with their associated probability (beta value) of belonging to that topic, the “tidytext” R library [36] was used.

## 3. Results

### 3.1. Descriptive Statistics

**Figure 3:**
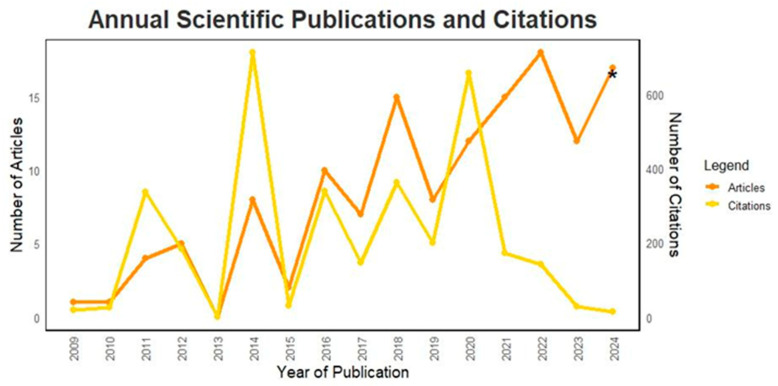
Annual publication and citation data for peer-reviewed papers published within the period 2009–2024 (September).

The research resulted in a total of 135 papers published between 2009 and 2024 (up to September, S1). Figure 3 illustrates the annual trend of publications related to our topic, alongside the number of citations per year. Publications on this topic began in 2009 with only one paper. The peak year for publications was 2022, with a total of 18 papers; however, 2024 may still exceed this number, as 17 papers have already been published. In terms of citations, the year 2014 recorded the highest total, with 711 citations.

**Figure 4:**
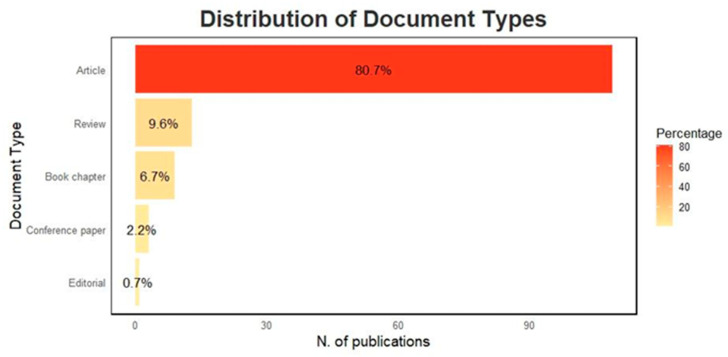
Bar graph illustrating the distribution of the 135 papers by document type.

Figure 4 presents the distribution of papers by document type. The most common document type is the Article, with 109 papers published (80.7%). This is followed by the Reviews, which account for 13 papers (9.6%), and Book chapters, with 9 papers (9.6%). Conference papers comprise three papers (2.2%), and Editorial books make up the smallest category with one paper (0.7%).

**Figure 5:**
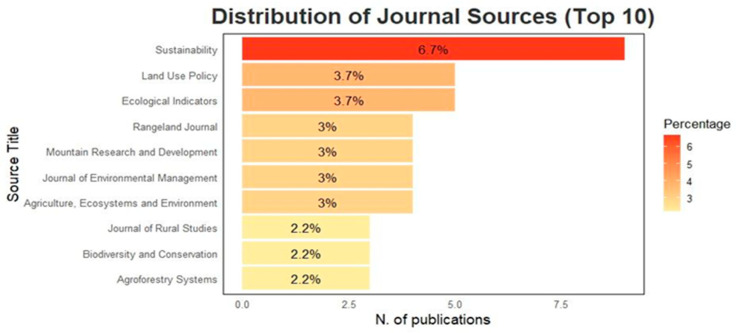
Bar graph of the top 10 journals by percentage of published papers.

Figure 5 shows the first 10 journals in which documents relating to our topic were published. The journal with the most publications is *Sustainability,* with nine papers (6.7%). This is followed by three journals with five papers each: *Land Use Policy* (3.7%) and *Ecological Indicators* (3.7%). There are also four journals with four papers each: *Rangeland Journal* (3%), *Mountain Research and Development* (3%), *Journal of Environmental Management* (3%), and *Agriculture, Ecosystems and Environment* (3%). Finally, three journals have 3 papers each: *Journal of Rural Studies* (2.2%), *Biodiversity and Conservation* (2.2%), and *Agroforestry Systems* (2.2%).

**Figure 6:**
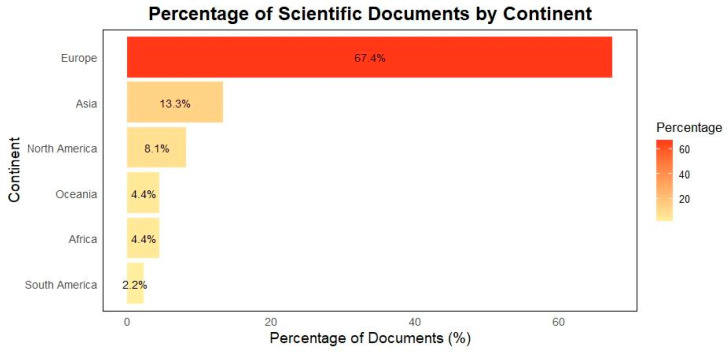
Bar graph of the distribution of 135 scientific literature records by continent.

Regarding the geographic area of origin of the publications, this was determined based on the nationality of the corresponding author and, where this information was not available, on that of the first author. The geographic distribution of these documents is as follows: Europe contributed 91 documents (67.4%), followed by Asia with 18 documents (13.3%). North America accounted for 11 documents (8.1%), Oceania and Africa for 6 documents (4.4%), and lastly, South America for 3 documents (2.2%). These data are illustrated in Figure 6.

**Figure 7:**
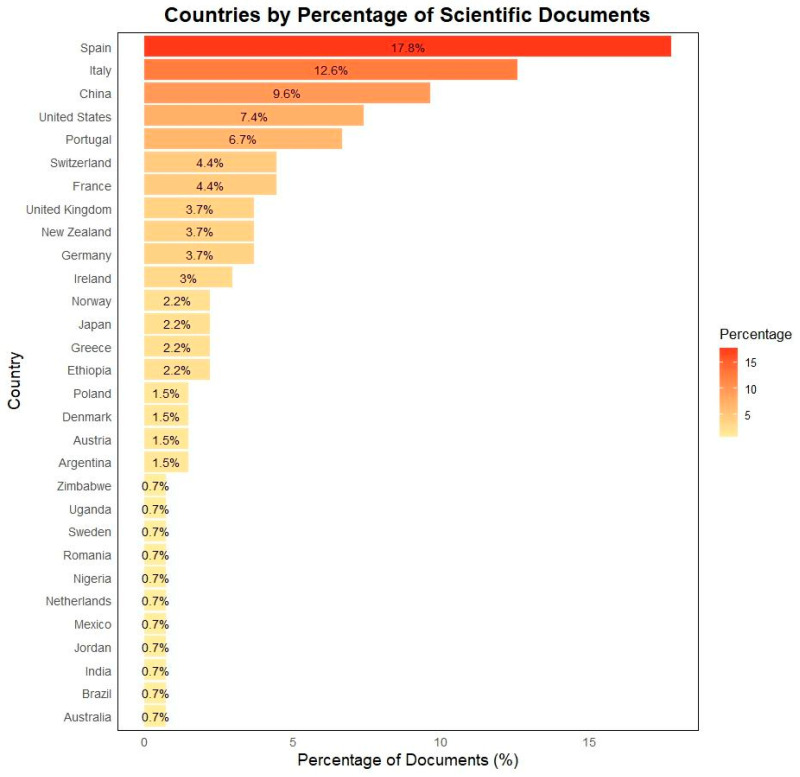
Bar graph of the distribution of 135 scientific literature records by countries.

A total of 28 countries contributed to the publication of papers (Figure 7). Spain led the count with 24 documents (17.8%), followed by Italy with 17 documents (12.6%) and China with 13 documents (9.6%). The United States and Portugal each contributed nine documents (6.7%). France and Switzerland each published six papers (4.4%), while Germany, New Zealand, and the United Kingdom each contributed five papers (3.7%). Ireland published four papers (3%), and Greece, Norway, Ethiopia, and Japan each produced three documents (2.2%). Argentina, Austria, Denmark, and Poland each contributed two papers (1.5%). Finally, Australia, Brazil, India, Jordan, Mexico, the Netherlands, Nigeria, Romania, Sweden, Uganda, and Zimbabwe each published one paper (0.7%).

**Figure 8:**
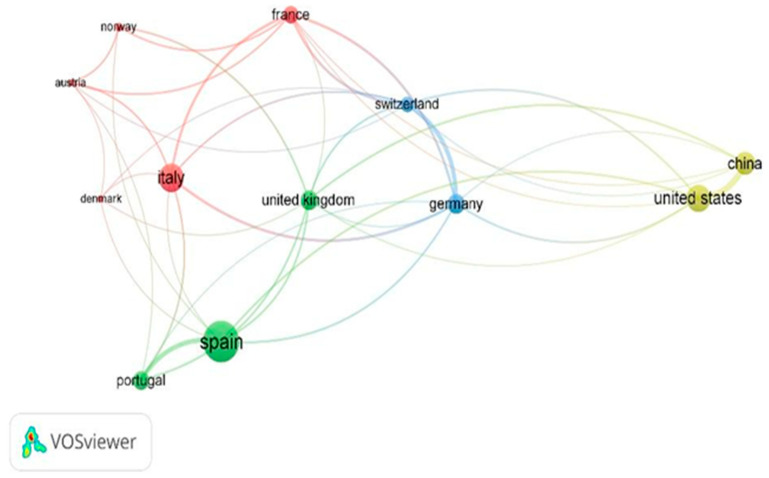
The network visualisation map of international research collaboration in the field of ES linked to extensive sheep and goat farming in mountain areas generated by VOSviewer [33], illustrating the collaborative landscape.

The rate of international cooperation is illustrated in Figure 8, reflecting the networking activity among researchers focused on the ES provided by small ruminants in mountainous regions, as obtained through VOSviewer. This analysis revealed twelve countries, listed in order of significance: Spain; Italy; United States; China; Germany; United Kingdom; Portugal; France; Switzerland; Norway; Austria; and Denmark. Countries that are located close to each other and linked by thick lines represent strong research partnerships. Conversely, countries on the periphery, with thin connecting lines to central nations, indicate limited collaboration. The font size reflects the volume of papers published in collaboration with other countries, highlighting the importance of these research connections. The colours represent the thematic clusters of the countries. Specifically, countries within the same cluster (indicated by the same colour) have collaborated more frequently with each other and are often co-authors in the same publications.

### 3.2. Insights from Text Mining

**Figure 9:**
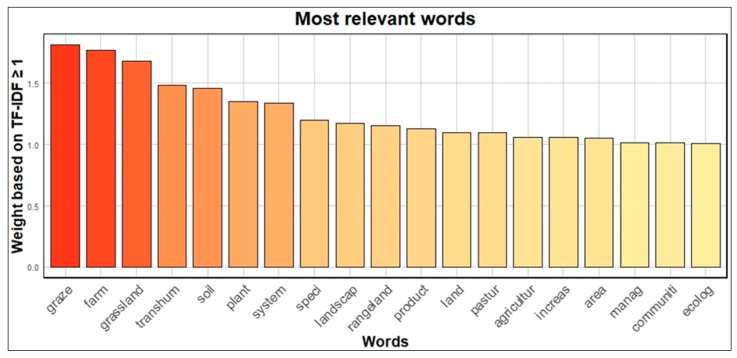
Graphical representation of the most relevant words (keeping only those with a weighting ≥ 1). Darker colour means heavier words, based on a term frequency–inverse document frequency (TF–IDF) technique.

**Figure 10:**
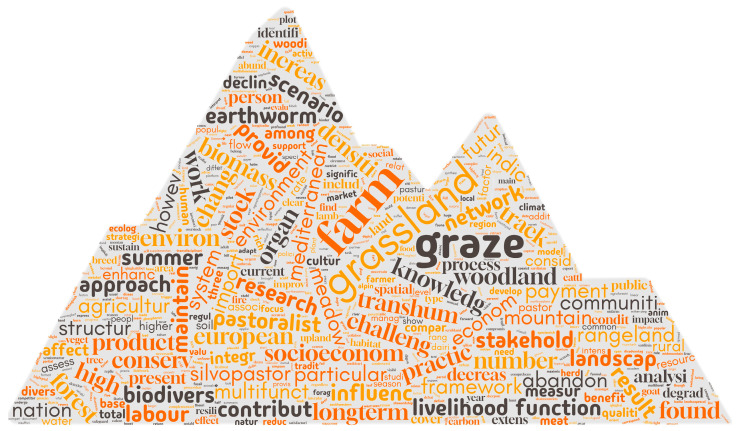
Cloud representation of the most relevant word stems (1418) in the database. The size of each term indicates its relative importance. This graphic was created using wordclouds.com (accessed on 31 October 2024) [32].

After pre-processing and the removal of sparse words, the final count of word roots was 1418. The term weights calculated using TF–IDF ranged from 1.83 to 0.05. The most relevant words, according to the TF–IDF weighting system, are displayed in a histogram and a word cloud (Figure 9 and Figure 10). A total of 19 words has a TF–IDF value greater than 1, including “graze”, “farm”, “grassland”, “transhum”, “soil”, “plant”, “system”, “speci”, “landscap”, “rangeland”, “product”, “land”, “pastur”, “agricultur”, “increas”, “area”, “manag”, “communiti”, and “ecolog”. Figure 10 displays a word cloud, where the size of each word reflects its significance, with font size proportional to the TF–IDF value of each term.

**Figure 11:**
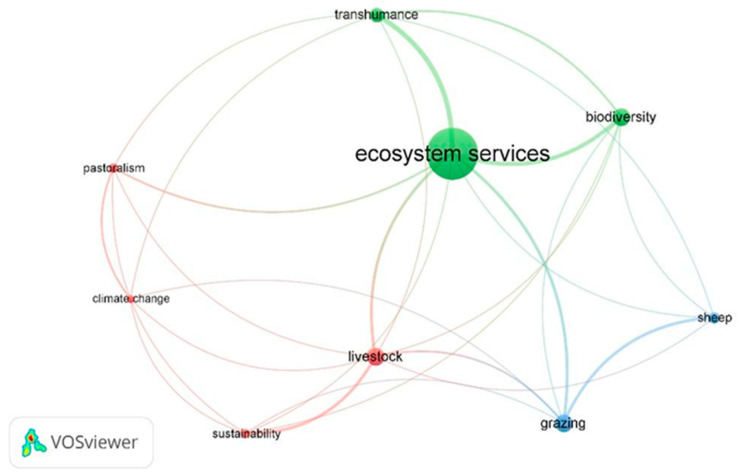
Co-occurrence network analysis of the top 9 keywords, generated by VOSviewer [33], organises the keywords into clusters (with a minimum cluster size of two items). The thickness of the connecting lines indicates the strength of their relationships, while the size of each node reflects the frequency of the respective keyword.

The co-occurrence network analysis, which visualises the relationships among the nine most frequent keywords in the collection, is presented in the maps of Figure 11. The colours represent the different clusters obtained through the analysis (three clusters). Specifically, green represents ecosystem services, biodiversity, and transhumance; blue represents grazing and sheep; red represents livestock, sustainability, climate change, and pastoralism. The size of each circular node in the map is proportional to the frequency of the keywords. The distance and thickness of the lines between words indicate the strength of their relationships. The most frequently occurring keyword is “ecosystem services”, with 33 occurrences, followed by “livestock”, “biodiversity”, and “grazing”, each with 11 occurrences. Additionally, “transhumance” appears with nine occurrences, “sheep” with eight, and both “sustainability” and “pastoralism” with six occurrences. Finally, “climate change” and “grassland” each have five occurrences.

### 3.3. Key Themes Emerging from Topic Analysis

**Figure 12:**
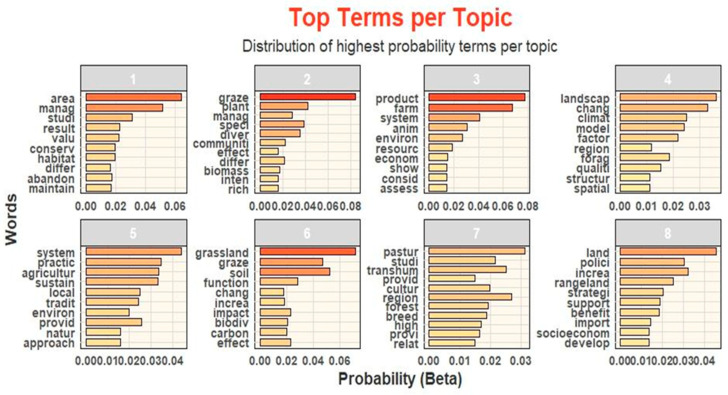
Most relevant 10 stem words per topic in the LDA (latent Dirichlet allocation) with 8 topics (beta = probability that a word belongs to a given topic).

The results of the TA are shown in Figure 12. Topic analysis highlighting eight main topics related to ES connected to extensive sheep and goat farming in mountain areas.

The most prominent is topic 2, which stands out with 20 publications. This topic, titled “Grazing effects on biodiversity”, is also the oldest, dating back to its first publication in 2009. Interestingly, most of the research in this area comes from Spain. Following closely are topics 3, 5, and 8, each with 18 publications. These cover a range of themes, which we labelled “Dimensions of sustainability”, “People engagement”, and “Policies and strategies”. Topic 7, with 17 publications, focuses on “Transhumance”, a theme where Italy takes the lead with seven contributions. Next, topics 4 and 6, each including 15 publications, are dedicated to “Climate” and “Grazing and soil”. In both cases, China emerges as the leading country in terms of research output.

Finally, topic 1, with 14 publications, is centred on “Ecosystem conservation” and is also closely tied to Spain, where most of the research originates.

All results are summarised in Table 2, where each topic is associated with potential ES and EDS. The ES was classified according to the Common International Classification of Ecosystem Services (CICES), which has a hierarchical structure based on five progressively more detailed levels, grouping the ES into three categories: provisioning; regulation and maintenance; and cultural [37]. No official classification is currently available for EDS [38].

## 4. Discussion

Our study aimed to investigate the scientific publications on ES linked to extensive sheep and goat farming in mountain areas, identifying 135 relevant papers through Scopus, published from 2009 onward. The earliest article, by Zhang and Dong [39], examined the effects of grazing to restore ecosystems and enhance biodiversity in the Zhongtiao Mountains, China. Since then, research interest in this topic has increased, with peaks and fluctuations in publication rates. Dong et al. [40], whose work is the most cited with 480 citations, contributed significantly with a study on the Qinghai–Tibetan Plateau, China, addressing ecosystem degradation and proposing adaptive livestock management strategies. China is in Asia, the continent with the highest concentration of small ruminants globally, according to the FAO data [13] and ranks second in the number of publications on this topic, with Europe leading the field. This trend can be attributed to the vast mountainous regions in Asia, where small ruminant farming is a prevalent practice. Europe’s dominance in publication numbers reflects a significant research focus on livestock farming topics. This is supported by Zuliani et al. [26], which highlights Europe’s collaborative research networks, particularly among neighbouring countries like Spain, Portugal, and France. Such collaboration, as revealed by VOSviewer analysis, underscores shared environmental and socio-economic interests, as also observed in studies on precision livestock farming by Marino et al. [21].

The journals in which these papers are published are predominantly of European origin and are well-established within their respective fields, boasting high-impact factors. Notable examples include Sustainability (IF: 3.0), Land Use Policy (IF: 6.0), and Ecological Indicators (IF: 7.0). These journals focus on the monitoring and assessment of ecological and environmental indicators, often in relation to management practices. They also cover a broad spectrum of interdisciplinary topics, including the social, economic, political, legal, and physical dimensions of both urban and rural land use planning.

### 4.1. Text Mining

The TM analysis revealed key terms (with TF–IDF > 1) such as “graze”, “farm”, and “grassland”, highlighting a strong research focus on grazing and land management in the reviewed publications. Other terms identified had a TF–IDF value below 1. Notably, the term “graze” had a particularly high weight (TF–IDF > 25) in the study by Zuliani et al. [26] on mountain livestock farming and, in turn, emphasised key terms like “sheep” and “goat”. Since the focus of our analysis was specifically on small ruminant farming in mountain areas, the terms “sheep” and “goat” were excluded from the search criteria as stop words. However, these terms appeared in the VOSviewer co-occurrence network analysis, identifying “sheep” as a relevant term across the 135 documents. The most significant term emerging from the VOSviewer analysis is “ecosystem services”, which is closely associated with transhumance and biodiversity. According to the Millennium Ecosystem Assessment (MEA) [41], ES are the benefits that people derive from ecosystems, encompassing both direct and indirect contributions to human well-being, most of which do not have a market value [42,43,44]. Small ruminant farming, managed through agroecological practices, plays a crucial role in providing ES, particularly in mountain areas, where grazing management supports biodiversity and ecological stability. While livestock farming drives rural landscapes and economies, its contribution to society is often overlooked and rarely quantified [45]. These benefits are captured in the concept of multifunctionality, which emphasises that livestock farming not only produces goods but also generates secondary positive effects like improved soil quality and landscape preservation [46,47]. In this context, ES highlights the importance of small ruminant farming in supporting both agricultural productivity and natural resource conservation.

However, it is important to acknowledge that poor livestock management can result in EDS, such as overgrazing, soil erosion, biodiversity loss, and greenhouse gas emissions (GHG) contributing to climate change [48]. Despite these impacts, there is no specific category for EDS in ES frameworks like MEA, CICES, or TEEB [38]. This gap limits the understanding of the full range of impacts associated with livestock farming. Integrating ES into small ruminant farming is essential for developing more sustainable management practices. Recognising and quantifying both the benefits and disservices of these systems is key to guiding policies that maximise ecological benefits while minimising environmental harm.

### 4.2. Relevance and Implications of Topic Analysis Findings

The application of the TA to scientific papers on ES related to extensive sheep and goat farming in mountain areas led to the identification of eight distinct topics that are discussed following the sequence obtained from the topic analysis, as shown in Figure 12.

#### 4.2.1. Ecosystem Conservation

Topic 1, accounting for 10.37% of the total documents, was labelled “Ecosystem conservation” based on an analysis of the key terms and thematic focus of the documents within this category. Prominent terms identified included “area”, “manag”, and “studi”. Conservation strategies within this topic focus on the habitat-level effect of small ruminant grazing to maintain these systems, often stressing the need for indicators to monitor ecological changes and guide efforts [49]. Grazing is one component of ecosystem conservation.

Traditional livestock farming, especially in Europe, has shaped semi-natural landscapes, creating habitats with high biodiversity and providing key ES, such as pollinator habitats and pest control [50]. For example, a Swedish study of 62,937 farms showed that small ruminant farms positively impacted landscape diversity, with small sheep farms significantly improving semi-natural grasslands (Cohen’s d = 1.3) and small-scale habitats (d = 0.27) [50].

Mountain regions offer critical ES but face challenges from rural abandonment, which leads to passive rewilding. This process enhances natural ecosystems but also reduces landscape heterogeneity and biodiversity, particularly in areas historically shaped by agro-pastoral practices [49,51,52]. Primi et al. [52] documented a 70-year transformation in the Italian Apennines, with an 18% decrease in animal units, a 22% reduction in open habitats, and a 15% increase in forest areas. This led to an 18% increase in patch aggregation and a decline in ecosystem complexity.

The loss of traditional management has also resulted in ecological connectivity for wild ungulates and carnivores, such as wolves, while reducing habitat suitability for species dependent on open meadows and ecotones [52,53]. Bueno et al. [49] observed that nearly 100% of high-pastoral-value grasslands in certain areas were impacted by wild boar rooting. Active habitat management is essential for maintaining semi-natural vegetation, hedgerows, and open landscapes, which are crucial for biodiversity and fire prevention [54]. Bernués et al. [55] highlighted grazing in semi-natural habitats as highly effective for soil fertility (4.5 ± 0.57, on a 0 to 5 scale) and biodiversity conservation (4.6 ± 0.69) in Mediterranean regions. Goat grazing has proven particularly effective in reducing shrub biomass for fire risk management. Studies in Iberia show that goats are three times more effective than sheep and twice as effective as cattle for this purpose [56].

Grazing also supports fire prevention programs in Mediterranean regions, where wildfires threaten mountain ecosystems [51,57]. For example, in Northern Portugal, goats grazing at densities of 1000 animals/ha for 2–4 days reduced shrub volume by over 50% in low-load plots, although regrowth occurred within six months [57]. Another study in Spain demonstrated that goats grazing at 2.7 animals/ha reduced shrub volume by 32%, increased bare soil by 51%, and decreased flammability by 22% [58]. Sustainable grazing densities vary locally, with annual averages of 0.45 ± 0.12 LU/ha in gorse shrublands and 0.33 ± 0.06 LU/ha in rock rose shrublands reported in the Iberian Peninsula [51].

However, both overgrazing and undergrazing present risks. Overgrazing leads to soil erosion and biodiversity loss, while undergrazing allows for biomass accumulation, increasing fire risks and altering habitat composition [57,59]. Balancing grazing pressure is crucial to avoid these outcomes and sustain ecosystem health.

While grazing can positively influence mountain ecosystems, rewilding initiatives promoting natural processes pose new challenges. For instance, the return of large carnivores like wolves has heightened human–wildlife conflicts, discouraging pastoral practices and rendering agri-environmental measures less effective [60]. Darnhofer et al. [54] employed a Delphi survey among experts in Austria, France, and Norway, highlighting the need for participatory and flexible agri-environment measures tailored to local conditions.

Balancing rewilding with traditional land use is critical for sustaining ES and fostering ecological and economic resilience. Future policies should integrate conservation with adaptable mixed land use, supported by iterative social learning processes and incentivized by Payments for Ecosystem Services (PES) [61].

#### 4.2.2. Grazing Effects on Biodiversity

Topic 2 has the highest number of publications, with 20 documents, accounting for 14.81% of the total. The main terms identified by the TA include “graze”, “plant”, “speci”, “diver”, and publications specifically focus on examining the effects of grazing on biodiversity.

Mountain grazing plays a pivotal role in preserving ecosystems of high ecological value, such as alpine grasslands protected under the European Habitats Directive [62]. By maintaining open habitats and promoting biodiversity, grazing supports both ecological stability and the livelihoods of mountain communities [16,63]. However, economic challenges and land abandonment pose significant risks to these landscapes, emphasising the need for sustainable management practices to safeguard their ES [64,65,66]. While land abandonment can promote natural succession, and rewilding is increasingly seen to restore ecological balance, the uncontrolled growth of shrub vegetation is a significant challenge. These include biodiversity loss, an increased risk of wildfires, and the disappearance of traditional landscapes [67]. The expansion of green alder (*Alnus viridis*) in European mountain pastures is an example. While this native species aids nitrogen fixation, its spread can lead to biodiversity loss, landscape changes, eutrophication, and increased GHG emissions. Historically, grazing, especially by goats, limited its spread, but the abandonment of grazing has made pastures more vulnerable [68]. Pauler et al. [68] stress that reintroducing small ruminants is key to mitigating these effects. In a 13.4-hectare pasture, partially dominated by *Alnus viridis* and with *Sorbus aucuparia* present, 15 paddocks were created, each with heterogeneous vegetation, varying forage quality, different ground inclinations, and varying degrees of *A. viridis* cover. Animals were rotated through the paddocks with an average stay of 12 days to simulate different grazing intensities. The average animal unit load densities (LU) were bovine: 0.515 LU/ha/year, sheep: 0.335 LU/ha/year, and goats: 0.21 LU/ha/year. Sheep were the most effective at controlling *A. viridis*, stripping an average of 244 branches per paddock (7.4% of total branches). Goats stripped 185 branches, but only 0.8% were from *A. viridis*, preferring *S. aucuparia* (82.3% of branches). Finally, cattle did not strip branches but only damaged shrubs by trampling and breaking branches. Small ruminants can, thus, serve as a barrier to invasive shrubs, helping to maintain open grasslands, conserve local biodiversity, and provide a sustainable model for managing mountain ecosystems.

Since 1960, in an area near Theix, France, mountain grasslands have been historically managed through grazing and mowing. In 2005, Hoeffner et al. [69] introduced five treatments to evaluate different management practices, replicated in four blocks: (i) mowing (MO); (ii) intensive grazing (CA+); (iii) low-intensity grazing (CA-); (iv) low-intensity sheep grazing (SH-); and (v) abandonment (AB). In CA- and SH- grasslands, epigeal abundance was 6.8 times higher than in mowed grasslands, with an overall increase in biomass and species richness of at least 1.9 times compared to abandoned grasslands. Abandonment led to a reduction in key species such as *Aporrectodea giardi* and *A. rosea*, highlighting the importance of active management.

However, Ważna et al. [70] demonstrated that grazing alters habitats by reducing biodiversity and encouraging opportunistic species, while practices such as mowing or abandonment increase biodiversity, offering valuable insights for sustainable ecological management of mountain environments. Their study examined the effects of sheep grazing on small mammal communities in the forest clearings of the Tatra Mountains, a protected alpine ecosystem. The glades, situated between 920 and 1174 metres above sea level and surrounded by fir trees and pines, have been managed with traditional practices or left to evolve naturally. Three types of use were studied: (i) non-managed glades (NG); (ii) glades mowed annually (MG); and (iii) grazing glades (GG), with a sheep density ranging from 4 to 17 individuals per hectare. The monitoring, conducted between 2004 and 2005, involved 2200 trap nights and used the capture–sign–recovery method to analyse small mammals. Data have shown that grazing, especially at higher densities (c.a. 15–28 sheep/ha vs. 5–9 sheep/ha), has a significant negative impact on biodiversity. In the grazing clearings, the number of species (7) was considerably lower than that of NG (13) and MG (11). The mean abundance was also lower (26 individuals per glade compared to 58 in NG). In GG, *Microtus arvalis* (common field mouse) dominated (70% of the individuals), while in NG and MG, co-dominance was observed among species such as *Microtus agrestis*, *Microtus subterraneus*, and other rodents (birch mice *Sicista betulina*).

The grazing of goats and sheep would seem to be a valuable tool for managing mountain landscapes, with complex implications for ES. Moderate grazing can enhance biodiversity by maintaining an ecological balance that supports the coexistence of diverse plant and animal species through controlled disturbances [71]. However, excessive grazing intensity, as highlighted by Ważna et al. [70], can reduce biodiversity by favouring opportunistic species such as *Microtus arvalis* and degrading more complex habitats. The resulting loss of biodiversity in grazed grasslands, compared to fallow or abandoned areas, underscores how poorly balanced management can undermine the ecological benefits and services provided by mountain grasslands. Grazing’s impact on biodiversity can be affected by multiple factors, such as the type of livestock, grazing intensity, seasonality, and climate [72]. By integrating grazing into active and adaptive management strategies, as successful examples across Europe demonstrate, one can maximise the benefits for biodiversity and ecosystems while preserving the ecological, cultural, and economic value of these landscapes.

#### 4.2.3. Dimensions of Sustainability

Topic 3, named “Dimensions of sustainability”, contains 13.33% of total documents, focusing on the sustainability of small ruminant production in mountain systems. The main terms identified by the TA include “product”, “farm”, “system”, “anim”, “environ”.

When addressing sustainability, it is essential to mention the 2030 Agenda, signed in 2015 by the member states of the United Nations for Sustainable Development, which comprises 17 goals (SDGs) aimed at protecting the planet, eradicating poverty, and ensuring peace and prosperity for all citizens [73]. Environmental [74], economic [75], and social aspects [76] are analysed through multi-level sustainability frameworks [77], with bioeconomy, circular and green economies at the core [78]. Social Life Cycle Assessment (S-LCA) evaluates social impacts and product contributions to SDGs [76].

Small ruminant farming produces meat, milk, skins and wool [79] but also generates environmental impacts such as GHG emissions, prompting monitoring initiatives [80]. O’Brien et al. [81] used Life Cycle Assessment (LCA) analysis, both in lowland and upland farms, to evaluate the environmental effects of intensifying pasture-based sheep farming, examining resource use, and environmental indicators such as carbon footprint, acidification, and eutrophication. Their study showed that increasing livestock production intensity could reduce emissions and resource use per kilogram of product [82,83]. Local environmental impacts on water and air quality remained within EU nitrogen and phosphorus limits [84], allowing for potential increases in national sheep production if pollution sources were managed effectively [81]. Improved pasture management, such as moving animals to nutrient-rich pastures during breeding, was recommended over reliance on concentrates [85], but upland farms, while having the smallest carbon footprint, were found to be the least efficient with the highest environmental impact.

Moreover, Cerrato et al. [86] used the LCA to evaluate the environmental and economic performance per livestock unit of three farming systems in the Cilento, Vallo di Diano, and Alburni National Park: sheep and goats only; cattle only; and mixed systems. Among the three farms, two were located in mountainous areas, while one was situated in a hilly area. All farms operated semi-extensively, with animals grazing outdoors except during particularly cold or rainy days. Their study showed that, economically, the cattle farm was the most profitable, yielding a net income of EUR 5427, followed by the mixed system with EUR 3154, and finally, the sheep and goat farm with EUR 473. However, the cattle-only farm showed the highest climate-warming impact, whereas the sheep and goat farm was the most environmentally sustainable. When considering all environmental impact categories, the mixed farm was the most sustainable overall, with lower emissions compared to the other systems [86]. This study highlights the environmental benefits of mixed systems but also emphasises the economic challenges associated with more sustainable practices.

Finally, the LCA can be integrated with the animal welfare analysis, considering both environmental impact and farm management practices. Using this integrated approach, Geß et al. [87] conducted a study on two farms in Northern Italy that produce lamb meat: the Educational Farm of the Department of Veterinary Sciences at the University of Turin, where Biellese lambs are semi-intensively managed and a farm in Val Maira (1700–2000 m a.s.l.), where Sambucana lambs graze on permanent pastures. The LCA assessed the environmental impact of products across their life cycle (ISO 14040; ISO 14044) [88,89], while cortisol analysis—a stress hormone found in wool—was used to evaluate the animals’ health.

The results demonstrated that while the more intensive farm showed lower impacts on global environmental factors, the extensively managed farm had higher animal welfare, reflected in lower cortisol levels. During the two data-collecting periods, cortisol concentrations in the semi-intensive system ranged from 7.74 ± 3.30 to 14.87 ± 4.51 pg/mg, whereas in the alpine system, levels varied from 8.32 ± 6.84 to 10.84 ± 4.92 pg/mg [87]. These findings emphasise the necessity of a balanced approach in livestock systems, where reducing environmental impact and ensuring animal well-being must coexist.

#### 4.2.4. Climate

Topic 4 accounts for 11.11% of total papers and includes key terms such as “landscap”, “chang”, “climat”, “model”, and others. Titled “Climate”, it explores the role of small ruminant farming in mountain regions in both mitigating climate change and adapting to its impacts. These farming systems play a vital role in key ecosystem processes, including carbon sequestration, soil stability, water regulation, and biodiversity conservation—topics also addressed in greater detail in Topics 1, 2, 3, and 6. However, the effectiveness of small ruminant farming in climate regulation depends on grazing practices, land management strategies, and adaptive responses to climate variability.

Climate change, resulting from the steady rise in GHG concentrations, represents one of the most pressing challenges facing humanity today [90]. According to a FAO report [91], small ruminants contribute 7% of the GHG emissions from livestock farming, which account for 12% of all anthropogenic GHG emissions. Among these gases, methane has the greatest impact on the GHGs produced by the livestock sector and is mainly produced through ruminal fermentation in ruminants [25,92]. Addressing these challenges requires the adoption of sustainable management practices, such as improvements in diet, rotational grazing, and adaptive grazing models, which can contribute to a better GHG balance [93,94,95,96].

Moreover, by promoting sustainable grazing practices, these systems contribute to “Regulation and Maintenance” ES, as shown in Table 2, such as long-term hydrological stability and ecosystem productivity, while also mitigating wildfire risks by reducing the accumulation of flammable biomass [97,98].

On the other side, climate change itself can also affect grazing livestock, particularly through droughts, reduced precipitation, and rising temperatures, which can alter pasture quality and quantity, as well as increase the incidence of pests and diseases [99,100,101]. These challenges can negatively impact both livestock welfare and productivity [102,103], leading to an increased methane emission per unit of product [15]. Therefore, identifying tolerant breeds with higher adaptive capacity to extreme environmental conditions is a viable strategy to mitigate the impact of climate change on small ruminant production [101].

#### 4.2.5. People Engagement

Topic 5 was labelled “People engagement” based on an analysis of the main words such as “system”, “practic”, “agricultur”, “sustain”, “local”, “tradit”, etc., as well as the general focus of the associated documents, which represent 13.33% of the total papers.

This topic explores the critical role of community engagement across various systems, emphasising its contributions to socio-economic impacts, cultural preservation, and participatory governance as pillars of socio-ecological sustainability. The sustainability of agricultural and pastoral systems, often located in ecologically fragile areas, depends on the active participation of local communities. By blending traditional practices with contemporary socio-economic needs, these systems achieve a dynamic balance that supports biodiversity, cultural heritage, and local livelihoods. Agricultural heritage systems, like those designated as Globally Important Agricultural Heritage Systems (GIAHS), significantly contribute to local economies by supporting livelihoods and promoting biodiversity. For instance, agro-silvo-pastoral landscapes generate income through ecotourism and high-quality traditional products, highlighting the multifunctionality of this system [104]. Similarly, Agri-Environment Climate (AEC) measures implemented in mountainous regions like Trento, Italy, align traditional agricultural practices with socio-ecological goals. These initiatives, which include payments for ES, support local economies by preventing land abandonment and promoting sustainable grazing systems [105]. In the Cévennes National Park, agro-pastoral activities sustain rural development through diversified outputs, including milk, meat, and traditional cheeses like Pélardon [106].

Despite these benefits, economic challenges persist. For instance, declining market demand for traditional products, such as hill lamb in the Irish uplands, underscores the dependence of these systems on subsidies and off-farm incomes [107]. Diversification strategies, including eco-tourism and direct sales, have been crucial in addressing these challenges in High Nature Value (HNV) mountain farming systems [108]. Traditional practices embedded in agricultural and pastoral systems play a vital role in preserving cultural heritage. For example, the dry-stone terraces in GIAHS systems not only enhance agricultural resilience but also symbolise cultural identity [109]. Similarly, transhumance practices in the Pyrenees and Greece reflect the deep connection between communities and their landscapes [110]. In Patagonia, traditional activities such as seasonal grazing routes and Criollo sheep rearing sustain biocultural diversity while fostering a sense of community identity [111]. In the Cévennes National Park, sylvo-pastoralism and transhumance intertwine ecological and cultural sustainability, contributing to cultural resilience [106]. However, modern pressures like urbanisation and climate change threaten these traditions. Adaptive strategies that integrate cultural preservation into sustainable development frameworks are essential to mitigate these challenges [112].

Active community participation is central to the resilience of socio-ecological systems. In Southern Tunisia, traditional irrigation systems exemplify the role of local knowledge in resource management [113]. Similarly, participatory governance in China’s “Retire Livestock, Restore Rangeland Project” demonstrates the importance of involving pastoralists in conservation initiatives to maintain ecological integrity [114]. Collaborative management frameworks in mountain grasslands and HNV farming systems further emphasise how local input strengthens resource management and decision-making, enhancing both ecological and economic sustainability [108,115]. Educational initiatives in areas such as the Cévennes National Park and Samothraki empower local stakeholders through continuous knowledge sharing, fostering long-term engagement and adaptive capacities [106,116]. Community engagement is essential for balancing ecological, economic, and cultural needs in agricultural and pastoral systems, and the integration of traditional knowledge with participatory governance ensures resilience against global challenges such as climate change and economic crises. Future policies must prioritise inclusivity, adaptive management, and cultural preservation to sustain these socio-ecological systems. The aspects of support policies will be explored further in Topic 8.

#### 4.2.6. Grazing and Soil

Topic 6 includes terms related to the influence of small ruminant grazing on mountain soil (e.g., “grassland”, “soil”, “graze”, “function”, and “impact”), which refer both to ES and EDS, accounting for 11.11% of the total articles.

Soil quality, defined by the dynamics of its properties and processes [117,118], is a crucial factor for the sustainability of the global biosphere and, in particular, for the resilience of forest systems [119]. However, the global impact of grazing on soil quality and ES remains a debated topic [120,121,122]. Livestock grazing management can have both positive and negative impacts on soil quality, depending on factors such as animal density, length of the grazing season, soil type (texture), soil moisture during grazing, etc. [123,124,125,126,127,128]. These factors are essential for balancing the ES that grazing provides, especially those related to soil quality and productivity. Among the ES provided by small ruminant grazing, the most significant are those classified as “Regulation and Maintenance”, which include processes such as nutrient cycling, soil fertility improvement, and vegetation control.

As reported by Fonseca et al. [122], extensive sheep grazing, as a land-clearing technique, improves soil fertility compared to mechanical clearing, particularly in the surface layer (0–5 cm). Grazing increases organic matter (a 30% increase compared to mechanical clearing), extractable phosphorus and potassium (ranging from medium values of 51–100 mg kg^−1^ to very high values of >200 mg kg^−1^), the sum of exchangeable bases (from 6.12 to 10.89 cmol kg^−1^), cation exchangeable capacity (from 6.6 to 11.4 cmol kg^−1^), and soil pH values (from 5.6 to 5.8). Furthermore, small ruminant grazing in mountain environments facilitates vegetation clearing and enhances soil productivity and environmental functions, including increased fertility and carbon sequestration [122].

Small ruminant grazing also impacts soil physical properties. Houlbrooke et al. [129] found that total soil porosity between 0 and 30 cm depth was lower in areas grazed by cattle compared to those grazed by sheep, indicating higher soil compaction. This study assessed total porosity under both irrigated and dryland conditions. In irrigated areas, soil porosity ranged from 46.6% to 55.2% for sheep and from 42.4% to 49.3% for cattle. In dryland areas, it ranged from 44.7% to 55.2% for sheep and from 45.6% to 51.9% for cattle. This difference is attributed to the higher static pressure exerted by a single cow (160–192 kPa) due to its greater weight per hoof area compared to a single sheep (83 kPa) [130]. Evidence from the United States further suggests that mixed grazing by sheep and cattle has less impact on soil properties than grazing them separately [131]. For instance, Jordon [132] found that introducing sheep into pastures previously grazed by only cattle reduces soil compaction over a summer grazing period (June to September).

Finally, extensive grazing in mountain pastures can influence soil microorganisms through plant biomass consumption, excretion input, and treading, with the magnitude of these effects being modulated by stocking density [133,134]. Garmendia et al. [135] stated that extensive mountain dairy sheep grazing systems have a positive effect on the soil microbial metabolic quotient, which measures soil microbial CO_2_ emissions (CO_2_ flux, expressed in g CO_2_ m^−2^ h^−1^) per microbial biomass (a measure of the mass of bacteria and fungi living in soil organic matter, expressed in mg microbial C kg^−1^ soil). This quotient provides valuable information about the efficiency of microbial metabolism. Compared to abandoned land, the soil microbial metabolic quotient was approximately 0.27 units lower in the grazing plots, indicating a more efficient microbial metabolism. This difference is due to the lower basal soil respiration (CO_2_ flux: 1.55 mg CO_2_ m^−2^ h^−1^ in exclusion plots vs. 1.32 mg CO_2_ m^−2^ h^−1^ in grazing plots) and the higher microbial carbon biomass (1315.0 mg microbial C kg^−1^ soil in exclusion plots vs. 1451.6 mg microbial C kg^−1^ soil in grazing plots). According to Aldezabal et al. [136], the lower microbial metabolic quotients in grazed plots are associated with reduced CO_2_ emissions and increased microbial biomass. This effect may be linked to higher soil temperatures observed in grazed plots [137], which also promote microbial biomass. In the absence of herbivores, an increase in microbial oxidative activity leads to an undesirable rise in CO_2_ emissions into the atmosphere from microbial respiration [135].

These results suggest that poorly managed grazing can degrade every ES into an EDS, converting what should be a source of ecological balance into a driver of environmental degradation.

#### 4.2.7. Transhumance

Topic 7, which includes 12.59% of the total documents, was labelled “Transhumance”, following an analysis of the main terms and the focus of the documents within it. Among the key terms, “pastur”, “region”, “transhum”, “studi”, and “cultur” were highlighted. Documents on this topic primarily explored the ES associated with sheep and goat transhumance across different regions, with Italy standing out as the country with the highest number of publications, as shown in Table 2. Transhumance, defined as the traditional seasonal movement of livestock between geographical or climatic regions [138], is a multi-millennial practice with evidence dating back to the Neolithic [139,140]. This practice allows for the efficient use of grazing resources over large spatial scales, maximising the use of natural grazing lands and taking advantage of phenological differences across areas with contrasting climate and seasonal patterns [141,142,143]. Transhumance can be categorised as either “vertical” (movement between mountain pastures and lower valleys) or “horizontal” (seasonal movement across long distances between different regions) [144,145]. The vertical type is characteristic of montane regions, where it plays a vital role in preserving cultural heritage and supporting socio-economic sustainability [146].

In recognition of its importance, the United Nations Educational, Scientific, and Cultural Organisation (UNESCO) in 2023 recognised both transhumance types as an “Intangible Cultural Heritage of Humanity”, celebrating a tradition deeply rooted in environmental knowledge and social practices [146]. According to Wolff [147], “transhumance is a multifunctional system anchored in a territory and a community that includes people and their natural environment with an interaction between the material and the immaterial in a socio-cultural practice”.

In the Mediterranean, herds are moved to upland regions during the cooler, well-watered summer months to avoid unpalatable dry vegetation and prevent overgrazing of lowland pastures. This practice is deeply tied to the social and spatial organisation of landscapes, with shepherds constructing pens and storage facilities or utilising natural shelters, such as caves and rock formations, for both livestock and themselves [140].

Although horizontal transhumance has largely been abandoned, daily vertical transhumance, even if dramatically decreased, persists in Piedmont, North Italy [148], and in areas like Prati di Mezzo in the Abruzzo, Lazio, and Molise National Park [149]. Here, small herds of sheep (Sopravissana, Massese, and Comisana breeds) mixed with goats (Grigia Ciociara, Capestrina, and Bianca Monticellana breeds) graze on biodiverse high-altitude pastures during summer months (June–October), covering daily routes of 7–10 km. This pastoral system, studied by Bindi et al. [149], provides a wide range of ES. It delivers provisioning services by supporting the production of meat, wool, milk, and traditional dairy products such as Pecorino di Picinisco (PDO), Marzolina (Slow Food Presidio), and other speciality cheeses. It also provides regulating and maintenance services, including enhancing soil fertility, promoting seed dispersal, reducing fire risk through the management of forage and woodland resources, as already explained in Topic 1, mitigating climate change with low CO_2_ emissions (3–8 kg CO_2_ eq. per kg of milk), and stabilising soil to prevent hydrogeological risks. In addition, this system preserves cultural heritage by fostering local traditions, strengthening community identity, and enabling cultural events such as the “Festa della Pastorizia” and the “Transhumanus” night walk along ancient routes. Furthermore, it supports biodiversity through the maintenance of semi-natural habitats and secondary grasslands, essential for sustaining autochthonous wild species such as the Abruzzo chamois (*Rupicapra pyrenaica*) and the Marsican bear (*Ursus arctos* ssp. *marsicanus*) categorised as vulnerable and critically endangered [150].

In this regard, there is a growing recognition that small ruminant farming in mountainous areas should no longer be viewed solely for its economic function but also its essential role in land management and conservation through the rational use of grassland and pasture resources [151]. Battaglini et al. [152] found that two summer grazing seasons with transhumant sheep on abandoned pastures in the Germanasca Valley (Turin) significantly reduced the invasive weeds and shrubs while improving forage quality. These results highlight the positive role of well-managed transhumant grazing in maintaining and restoring mountain areas.

Through its combined ecological, cultural, and economic contributions, daily vertical transhumance exemplifies a sustainable pastoral system that preserves landscapes, biodiversity, and cultural heritage [149]. Additionally, the recreational services associated with transhumance are gaining recognition, as drove roads increasingly serve as public spaces for recreational activities, environmental education, and local festivals celebrating this traditional practice [153,154]. In this way, transhumance offers an opportunity to discover pastoral culture and provides an additional source of income for local communities.

In addition to the various ES provided by transhumance, it is important to consider potential ED, such as dirtiness in some areas of the drove road that, as public spaces, might be misused as dumpers [155], or conflicts with modern land-use practices, etc. However, it is worth noting that the literature on EDS generated by transhumance is limited, highlighting the need for further investigation into this aspect.

#### 4.2.8. Policies and Strategies

Topic 8, accounting for 13.33% of the total documents, was labelled “Policies and strategies” based on an analysis of the key terms and thematic focus of the documents within this category. Prominent terms identified included “land”, “polici”, “increa”, “rangeland”, and “strategi”. The documents under this topic primarily examine strategies for maintaining ES associated with sheep and goat farming in mountainous regions, as they are increasingly at risk due to socio-economic shifts, land-use pressures, and climate change.

Reid et al. [156] noted that common policies aimed at enhancing resilience in mountain lands primarily focus on sustainable practices, such as community-based rangeland management (CBRM) and flexible tenure systems. For example, in Mongolia’s Altai Mountains, CBRM increased forage availability by 25% over five years. Similarly, in the Alps, collaborative grazing systems sustained livestock productivity while reducing soil erosion by 30% across monitored sites. However, mountain pastoral traditions are often integrated into regulations poorly suited to adaptive management, particularly when combined with additional environmental and health-related policies. This misalignment has contributed to the gradual abandonment of mountain areas, along with their associated livestock systems and biocultural heritage [52].

Protection policies for wildlife and ecosystems, in general, can affect sheep and goat mountain farming. Hendrickson [157] discusses how recent U.S. public land policies prioritise wildlife conservation over livestock grazing, threatening food security, rural economies, and 23% of the U.S. sheep industry. This shift removes grazing as a key tool for maintaining mountain rangeland health, controlling invasive species, and reducing wildfire risk. In Norway, carnivore management zones (CMZs), designed to balance carnivore conservation and livestock grazing, have reduced livestock losses in core areas, but conflicts persist within 50 km of their boundaries, impacting local farmers [158]. Mink et al. [159] analyse the impact of wolf attacks on Alpine summer farms in Switzerland, showing that frequent attacks accelerate the decline in vulnerable grazing systems, particularly for sheep. However, farming systems with better herd protection strategies show resilience and even increase in regions with frequent wolf presence. Gervasi et al. [160] estimated that in sheep farming across 10 European countries (2010–2015), four strictly protected species (i.e., wolves, wolverines, lynx, and bears) account for 45%, 24%, 19%, and 12% of compensated sheep kills, respectively—a figure that may seem relatively small on its own but does not account for the loss of ecosystem services due to the abandonment of pastoral practices as a consequence of predator presence. Stauder [161] identifies, in fact, a potential pressure of wolf presence on small ruminant farming as a driver for the abandonment of summer pasture grazing in the South Tyrol mountains of Italy, similar to Niedermayer and Gottschalk [162] in Germany. However, there is a notable lack of studies that associate and quantify the loss of ecosystem services potentially linked to this dynamic.

Extensive sheep and goat farming often operates at a financial loss in the absence of government subsidies. As Tsiouni et al. [163] highlighted, goat farms in Greece are not economically viable without external support, with a negative Net Farm Income recorded when subsidies are excluded.

Many European small ruminant farms face similar issues, where production costs frequently exceed total revenues due to a hostile production environment [164,165]. Galàn et al. [166] investigate the role of common lands in sustaining mountain grazing systems under the European Common Agricultural Policy (CAP), focusing on the Enirio–Aralar grazing commons in the Basque region of Spain. CAP payments, derived from Pillar 1 (direct payments and greening) and Pillar 2 (agro-environmental measures and natural constraint compensation), contribute significantly to farm profitability, accounting for 25% of total income and 54% of net margins. Payments associated with common lands alone represent 42% of net margins. Consequently, the sector remains heavily reliant on agricultural policy subsidies [167], highlighting their essential role in preventing the widespread abandonment of these livestock farming systems.

In this context, PES schemes have emerged as effective tools for incentivising sustainable practices in mountain pastoral systems. In general, these tools provide economic benefits by compensating herders for maintaining or enhancing ES while also aligning with broader environmental objectives, offering critical support to financially strained farming systems. Among the six main tools currently in use (e.g., direct public payments, direct private payments, tax incentives, cap-and-trade markets, voluntary markets, and certification programs), only a few have been investigated by researchers in the context of small ruminant systems in mountain areas.

Taube et al. [168] demonstrate that such initiatives can protect endemic species and high-value ecological zones. Hendrickson [157] emphasises the hydrological services provided by mountain pastoral systems, proposing that PES mechanisms can link upstream herders with downstream water users, fostering mutually beneficial conservation networks. Seid et al. [169] highlight that well-managed pastoral systems can act as significant carbon sinks, and PES schemes linked to carbon markets could provide financial rewards for implementing regenerative practices. In general, environmental and societal benefits associated with sustainable management practices of mountain small ruminant farming can be leveraged also through the emerging private ecosystem services market [170], but this topic is unexplored by the researchers.

To ensure the success of PES schemes, robust monitoring systems and equitable benefit distribution are essential. Advanced tools such as remote sensing and AI (artificial intelligence) can improve the precision of PES implementation by tying payments to measurable ecosystem outcomes, ensuring transparency and accountability.

Certification systems represent another avenue to sustain ES associated with sheep and goat pastoralism. These systems can transform the economic landscape for mountain pastoralists by creating market-driven incentives for sustainable practices. Rueff and Rahim [171] underscore the potential of region-specific branding, such as Geographical Indications for cheeses or wool, which capitalise on the unique ecological characteristics of mountain systems. Moreover, certifications tied to low-carbon practices or organic standards, as suggested by Taube et al. [168], attract environmentally conscious consumers, fostering a market premium for pastoral products. However, these certifications require coordinated efforts, including financial and technical support for producers, as well as the establishment of robust supply chains to effectively market certified and labelled goods [172].

Financial mechanisms, including subsidies, climate resilience funds, and PES programs, are indispensable for making sustainable pastoral practices economically viable. Hendrickson [157] advocates for integrating pastoral systems into broader socio-economic frameworks, such as rural development plans and national conservation strategies, to amplify their impact. Collaborative governance structures also play a critical role. Multi-stakeholder platforms involving governments, NGOs (non-governmental organisations), and private actors can design integrated policies that align agricultural productivity with ecological sustainability, ensuring that pastoralists are active participants in decision-making processes.

Modern strategies for managing mountain pastoral systems are underpinned by technological advancements. AI-driven image analysis and remote sensing tools, as discussed by [169], provide valuable insights into grazing intensity, vegetation health, and overall ecosystem performance. Additionally, blockchain technology can enhance transparency and trust in PES and certification programs, streamlining transactions and ensuring accountability.

By integrating PES schemes and credit markets, certification systems, and adaptive management practices, the long-term sustainability of mountain pastoral systems can be ensured. Targeted financial support, collaborative governance, and technological innovation will be essential to maximise their potential to deliver critical ES while preserving the ecological and cultural integrity of mountain regions.

## 5. Limits of the Methodology

The method used in this study has some limitations. Firstly, the search was restricted to the Elsevier database Scopus^®^, excluding papers from other databases and the “grey literature”. Additionally, not all the synonyms of key terms were considered in this methodology. Additional restrictions included the application of filters to limit the search to English-only abstract language or specific subject areas. Papers without accessible abstracts were also excluded, which may have further reduced the number of records included in this analysis.

Furthermore, text mining depends on the quality of the data, and misinterpretations can arise due to the algorithms’ inability to fully capture context. Topic analysis, on the other hand, may lack granularity, with broad or overlapping topics affecting the clarity of the results. Additionally, the labelling of topics is subjective, and the performance of the algorithms is influenced by their settings, which may not always capture the full complexity of the data.

Despite these constraints, this study provides a comprehensive overview of the literature on ES delivered by extensive small ruminant farming in mountainous areas, highlighting key topics associated with this livestock system.

## 6. Conclusions and Recommendations for Future Research

This study, utilising the innovative methods of TM and TA, highlights the growing scientific interest in ecosystem services associated with small ruminants in extensive mountain farming systems. Despite its novelty—evidenced by the first publications emerging only in 2009—this field is rapidly evolving, with Europe playing a leading role in research despite the global significance of small ruminants, particularly in Asia and Africa.

Key findings reveal the critical role of small ruminant farming in promoting sustainability, conserving biodiversity, and supporting rural livelihoods. However, gaps in the current literature, such as the insufficient evaluation of ecosystem disservices and the lack of standardised frameworks for assessing and inventorying ecosystem services, underscore the need for more comprehensive studies.

This analysis underlines the need for a holistic perspective on small ruminant farming, which recognises its potential to contribute to climate change mitigation, agricultural biodiversity, and socio-economic sustainability, particularly in mountain regions.

In our opinion, future research should prioritise the development of standardised, universal frameworks for assessing ecosystem services and disservices to harmonise evaluations globally. These frameworks should also include a comprehensive inventory of ecosystem services to provide a clear baseline for evaluation and monitoring. Incorporating innovative technologies can further improve accuracy, transparency, and scalability. Equally important is the design of effective certification systems to promote sustainable practices, enhance marketability, and provide tangible benefits for small-scale farmers. These schemes should be accessible, context-sensitive, and transparent, fostering trust among producers and consumers.

Refining grazing practices is another critical area for investigation, requiring long-term studies to identify region-specific strategies that maximise ecosystem services while minimising negative impacts. This includes exploring the ecological trade-offs of varying grazing intensities, seasonal patterns, and multi-species livestock farming under diverse environmental conditions. Additionally, integrating advanced technologies like AI, machine learning, and precision sensors into livestock management will enable real-time monitoring of animals, vegetation, soil health, and biodiversity. Such tools must be cost-effective and user-friendly, especially for small-scale pastoralists, and designed to engage younger generations.

Further research is needed to better understand the carbon sequestration potential of grazing systems and to optimise livestock diets for reducing methane emissions. Identifying resilient livestock breeds capable of adapting to extreme environmental conditions is also crucial to mitigating the impacts of climate variability on farming systems.

On the socio-economic front, evaluating the effectiveness of policy instruments such as PES programs and financial incentives is essential for supporting sustainable pastoral systems. Equally important is the assessment of ecosystem services in relation to specific territories and socio-economic contexts to ensure fair and effective compensation mechanisms. Additionally, investigating the role of cultural heritage in shaping pastoral practices can ensure the preservation of biocultural landscapes while meeting modern socio-economic development needs.

Finally, participatory frameworks that actively involve local communities in co-designing management practices are indispensable. Combining traditional ecological knowledge with scientific advancements will ensure practical, culturally appropriate solutions that foster community ownership and long-term sustainability.

Addressing these research needs will require collaborative efforts across disciplines, regions, and stakeholders.

## Figures and Tables

**Table 1:** Search strings for bibliographic analysis focused on TM related to the ES linked to extensive sheep and goat farming in mountain areas, applied to titles, abstracts, and keywords from the peer-reviewed English literature published from 1980 to September 2024.

Search Keywords	N. of Published Documents
“Ecosystem services” AND “mountain”	2155
“Ecosystem services” AND “ruminants” AND “mountain”	6
“Ecosystem services” AND “small ruminants”	13
“Ecosystem services” AND “ruminants”	94
“Ecosystem services” AND “sheep” OR “goat”	252
“Ecosystem services” AND “pastoral”	303
“Ecosystem services” AND “mountain livestock”	8
Total	2831

**Table 2:** Topic number, name of each topic, number of publications per topic, year of first publication, and the country with the highest number of publications from 135 documents.

N.	Theme	Papers (n)	Year of First Publication	Country	Ecosystem Services	Ecosystem Disservices
1	Ecosystem conservation	14	2011	Spain (5)	Provisioning, Regulation, and Maintenance	Habitat degradation; Biodiversity loss
2	Grazing effects on biodiversity	20	2009	Spain (4)	Regulation and Maintenance	Worsening of pasture quality; Weed spread; Biodiversity loss
3	Dimensions of sustainability	18	2012	German, Spain (3)	Provisioning	Environmental impact
4	Climate	15	2011	China (4)	Regulation and Maintenance	Environmental impact
5	People engagement	18	2014	Ethiopia (3)	Regulation and Maintenance	Interference with other human activities and wildlife
6	Grazing and soil	15	2012	China (3)	Regulation and Maintenance	Soil erosion and degradation; Biodiversity loss
7	Transhumance	17	2010	Italy (7)	Provisioning; Regulation and Maintenance; Cultural	Interference with other human activities and wildlife
8	Policies and strategies	18	2014	China, Ethiopia, Italy, United States (3)	-	-

## Data Availability

The original contributions presented in this study are included in the article/Supplementary Materials. Further inquiries can be directed to the corresponding author.

## Supplementary Materials

The following supporting information can be downloaded at: https://www.mdpi.com/article/10.3390/ani15030350/s1, Table S1: final dataset used for text mining and topic analysis.
